# Alcohol and the Adolescent Brain: What We’ve Learned and Where the Data Are Taking Us

**DOI:** 10.35946/arcr.v42.1.07

**Published:** 2022-04-07

**Authors:** Susan F. Tapert, Sonja Eberson-Shumate

**Affiliations:** Department of Psychiatry, University of California San Diego, La Jolla, California

**Keywords:** alcohol, adolescence, binge drinking, neuroimaging, magnetic resonance imaging, neuropsychological tests, young adults, drinking behavior

## Abstract

This article is part of a Festschrift commemorating the 50th anniversary of the National Institute on Alcohol Abuse and Alcoholism (NIAAA). Established in 1970, first as part of the National Institute of Mental Health and later as an independent institute of the National Institutes of Health, NIAAA today is the world’s largest funding agency for alcohol research. In addition to its own intramural research program, NIAAA supports the entire spectrum of innovative basic, translational, and clinical research to advance the diagnosis, prevention, and treatment of alcohol use disorder and alcohol-related problems. To celebrate the anniversary, NIAAA hosted a 2-day symposium, “Alcohol Across the Lifespan: 50 Years of Evidence-Based Diagnosis, Prevention, and Treatment Research,” devoted to key topics within the field of alcohol research. This article is based on Dr. Tapert’s presentation at the event. NIAAA Director George F. Koob, Ph.D., serves as editor of the Festschrift.

The past 50 years of research supported by the National Institute on Alcohol Abuse and Alcoholism (NIAAA) have resulted in an accumulation of invaluable data to address the multifaceted problems surrounding underage drinking. Youth use of alcohol remains a pervasive social and public health concern in the United States and a leading cause of disability and mortality during adolescence.^1^,^2^ Alcohol use in adolescence has a distinct pattern from adult drinking, whereby adolescents may have fewer drinking occasions but consume relatively high levels per occasion, referred to as binge or heavy episodic drinking and defined as consuming four or more standard ethanol consumption units on an occasion for females and five or more for males.^3^–^5^ Highly prevalent among youth in Western countries is an intermittent pattern of heavy alcohol consumption that typically is associated with social leisure occasions on weekend nights.^6^ Moreover, adolescent alcohol use, along with smoking and illicit drug use, has undergone changes in prevalence and patterns in recent decades. For example, alcohol use peaked in the mid-1990s, with approximately 50% of 12th graders reporting past-month alcohol use, followed by a steady long-term decline to 30% in 2018. In 2020, the downward trend reversed course, with 34% of 12th graders reporting past-month alcohol use.^1^ Recent reports indicate that prevalence estimates for 2021 will need to account for impacts of the COVID-19 global pandemic on underage substance use behavior and availability.^7^

High-risk alcohol consumption patterns and associated problems alone increase risk for adverse outcomes—such as motor vehicle accidents, high-risk sexual behaviors, other illicit substance use, and mental health challenges—for adolescents who drink. These risks are further compounded by the fact that adolescence is a period of crucial brain development and maturation.^8^,^9^ Neuroimaging studies have provided clear evidence that the brain (a) continues to develop throughout adolescence and into adulthood, and (b) undergoes important structural and functional changes in synaptic plasticity and neural connectivity during adolescence.^10^,^11^ These changes and the enormous plasticity of the teen brain make adolescence a time of both great risk and great opportunity.^11^

This article begins with an overview of typical adolescent brain development, followed by a summary of four key themes in the current understanding of alcohol and the adolescent brain: (1) predictors of underage drinking; (2) consequences of alcohol on adolescent brain structure and function; (3) moderating and confounding factors, including age of onset, sex disparities, family history, co-use of other substances, and mental health comorbidities; and (4) reversibility of and recovery from alcohol misuse. The article concludes with a discussion of where the data lead us to reach the next milestones in NIAAA-supported research.

## Typical Adolescent Brain Development

The brain of an adolescent, much like teenage behavior, undergoes significant developmental changes. This neurodevelopment continues after adolescence, typically until around age 25.^12^–^15^ The maturational processes in the brain occur in stages, with more basic functions (e.g., motor and sensory functions) maturing first and areas such as the lateral temporal and frontal lobes, which are responsible for higher cognitive function (e.g., decision-making, attention), developing later in adolescence.^13^ The prefrontal cortex is one of the last brain regions to complete its maturation. Its rate of change does not plateau until the third decade of life, in concert with typical developmental trajectories of cognitive abilities, such as decision-making, attention, and cognitive control.^16^–^18^ The late maturation of the prefrontal cortex has been linked to risky behavior during adolescence, particularly if the limbic subcortical system develops earlier.^16^

Executive functioning typically matures during this developmental stage,^19^ coincident with gray matter reductions and white matter growth.^20^,^21^ Functional magnetic resonance imaging (fMRI) studies of executive behaviors have demonstrated increasing prefrontal activity and better inhibitory control with adolescent age.^22^ Challenges in executive functioning have been observed in adolescents with a family history of alcohol use disorder (AUD),^23^ repeated childhood trauma experiences,^24^ and poor sleep,^25^ all of which also have been identified as risk for adolescent binge drinking and AUD.^17^,^26^,^27^ Deficits in control circuitry have been linked to impulsivity, sensation seeking, and alcohol use into early adulthood.^28^

One of the studies investigating adolescent alcohol use and its effects is coordinated by the National Consortium on Alcohol and Neurodevelopment in Adolescence (NCANDA), which is conducting a multisite longitudinal study supported by funding from NIAAA and other National Institutes of Health partner institutes. Launched in 2012, this five-site consortium recruited a community cohort of 831 diverse adolescents ages 12 to 21 from five U.S. regions (Durham, North Carolina; Palo Alto, California; Pittsburgh, Pennsylvania; Portland, Oregon; and San Diego, California). Half the sample was enriched for key characteristics conveying risk for heavy drinking among adolescents (i.e., family history of substance use disorder, youth externalizing or internalizing symptoms, and having tried alcohol by age 14). Most of the sample (85%) reported very limited alcohol use at project entry; the remaining 15% exceeded typical age thresholds for alcohol at project entry in this cohort-sequential design.^29^ At project entry and annually thereafter, participants received neuroimaging (high-resolution structural, diffusion, and resting-state fMRI scans), neurocognitive testing, detailed substance use and mental health interviews; provided urine samples for drug testing as well as saliva samples for genetics and pubertal hormone assays; and completed various self- and parent reports on personality, behaviors, and environment.^29^ NCANDA will continue to examine the interactive effects of typical development as well as adolescent alcohol use and executive dysfunction into early adulthood.

Resting-state fMRI findings from NCANDA and other studies have shown that intrinsic functional networks subserving cognitive control and limbic circuitry develop across adolescence and may be influenced by adolescent heavy drinking.^24^,^30^,^31^ Moreover, the adverse effects of alcohol may be more prominent in girls than in boys.^32^

## Predictors of Underage Drinking

Being able to identify youth at higher risk for alcohol misuse could lead to early intervention and ultimately help reduce the significant personal and public health burden of AUD; however, relatively few studies have explored individual-level precursors of adolescent alcohol use. Prospective longitudinal studies of substance-naïve youth are uniquely positioned to identify factors predating the onset of alcohol use. Squeglia et al. identified several markers of alcohol initiation by age 18 in 137 adolescents.^27^ These markers included demographic and behavioral factors (e.g., male sex, higher socioeconomic status, early dating, more externalizing behaviors, positive alcohol expectancies), lower executive functioning, thinner cortices, and less brain activation in diffusely distributed brain regions.

NCANDA seeks to expand on these findings using a greater number of measurements in a large sample to lead to more accurate individual-level forecasting. The consortium is employing machine learning models, which can avoid multiple-comparison correction and reduce measures to a single, individual-level prediction.^33^,^34^ NCANDA developed a model that distinguished youth who drink heavily from those who drink little or no alcohol, based on patterns of macrostructural and microstructural imaging metrics in multiple brain regions.^35^ The analyses suggested delayed development of white matter connectivity among the older youth in the sample who drank heavily, as well as increased risk of subsequent heavy drinking in youth with more externalizing symptoms. These findings fit closely with those from the IMAGEN Consortium, which found that variability in personality, cognition, life events, neural functioning, and drinking behavior features predicted Alcohol Use Disorders Identification Test scores at ages 14 and 16.^36^

## Neural Consequences of Underage Heavy Drinking

### Gray Matter Volume

Unlike white matter, gray matter volume peaks in the primary school-age years, around age 10.^11^ Squeglia et al. reported that youth who drank heavily (*n* = 75) (defined using modified Cahalan quantity × frequency criteria^37^,^38^) showed accelerated reductions in gray matter volumes in cortical lateral frontal and temporal areas compared to those who drank no or little alcohol (*n* = 59).^39^ These results were largely unchanged with co-use of marijuana and other drugs; also, similar patterns of developmental trajectory abnormalities existed in males and females. This finding was replicated in the NCANDA cohort, which examined the influence of alcohol use on gray matter structure in 483 adolescents ages 12 to 21 both before and 1 to 2 years after the onset of heavy drinking.^13^ For youth with no or low alcohol consumption, gray matter volumes declined throughout adolescence, with rates slowing in many brain regions in later adolescence. However, youth who initiated heavy drinking exhibited a steeper decline in frontal gray matter volumes. For both youth with no or low alcohol consumption and those with heavy drinking, cannabis use did not influence gray matter volume trajectories.

These findings were confirmed in a recent analysis spanning five time points in the NCANDA study and using linear mixed-effects models.^40^ A greater number of past-year binge drinking episodes was linked to greater decreases in gray matter volumes in 26 of 34 bilateral Desikan-Killiany cortical parcellations tested. The strongest effects were noted in frontal regions as well as among younger adolescents; moreover, the effects largely attenuated in later adolescence. The gray matter volumes decreased most for individuals with greater numbers of binge-drinking episodes and recent binge drinking. These findings provide yet more evidence that adolescent binge drinking is linked to a greater risk of more prominent gray matter reductions during adolescence.^40^

Functional MRI studies further suggested that adolescents with histories of heavy drinking showed aberrant patterns of activation in response to cognitively challenging tasks,^41^,^42^ including tasks of working memory and inhibition. In adolescents with a history of 1 to 2 years of heavy drinking, the aberrant activation was not linked to detectable deficiencies in task performance. However, if heavy drinking persisted longer, reduced task performance was often evident in the adolescents.^43^,^44^ This pattern of results suggested that the brain may be able to compensate for subtle neuronal insults for a period of time, but if drinking patterns persist and become heavier, the brain may no longer be able to compensate and may be vulnerable to the effects of repeated and sustained heavy doses of alcohol.

### White Matter Volume and Integrity

Throughout adolescence, white matter volume increases and matures, resulting in myelination that increases speed of neuronal transmission and modulates the timing and synchrony of neuronal firing patterns that convey meaning in the brain.^11^ Squeglia et al. reported that adolescents who drank heavily showed attenuated white matter growth of the corpus callosum and pons relative to adolescents who did not drink.^39^ Pfefferbaum et al. indicated that among those in the NCANDA sample who consumed no or little alcohol, white matter regions grew at faster rates in younger age groups and slowed toward young adulthood.^13^

To examine the potential for a neurotoxic effect of alcohol use on adolescent development of white matter, Zhao et al. conducted a whole-brain analysis of fractional anisotropy of NCANDA participants ages 12 to 21 at baseline.^45^ For 63 adolescents who initiated heavy drinking, the researchers examined white matter quality before and after drinking onset and compared it to the white matter maturation trajectory of 291 adolescents with no or low alcohol consumption. Results showed deterioration of white matter integrity in youth who drank heavily compared with age- and sex-matched controls. Moreover, the slope of this reduction over time corresponded with days of drinking since the study entry.^45^ Within-subject analyses contrasted developmental trajectories of youth before and after they initiated heavy drinking. These analyses suggested that drinking onset was associated with, and appeared to precede, disrupted white matter integrity. This disruption was greater in younger adolescents than in older adolescents, and was most pronounced in the genu and body of the corpus callosum.^45^ It is possible that these brain structure changes may occur concomitantly with modifications in certain neurotransmitter and hormone secretion systems, which markedly influence the refinement of certain brain areas and neural circuits.^46^

### Neurocognition

Along with altered development and maturation of gray and white matter, studies have reported neurocognitive consequences of underage drinking, such as impairments in attention,^47^ verbal learning,^48^,^49^ visuospatial processing,^47^,^50^ and memory.^49^ Neurocognitive deficits linked to moderate to heavy drinking during this critical developmental period may lead to direct and indirect changes in neuromaturational course, with effects that may extend into adulthood. Squeglia et al. examined neurocognitive function in adolescents who drank heavily, moderately, or not at all, based on the Cahalan classification system.^51^ Their findings suggested that initiation of moderate to heavy alcohol use and incurring hangovers during adolescence may adversely influence neurocognitive functioning. For females, more drinking days in the past year predicted a greater reduction in performance on visuospatial tasks, in particular visuospatial memory, from baseline to follow-up. For males, a tendency was seen for more hangover symptoms in the year before follow-up testing to predict a relative worsening of sustained attention.^51^

### Alcohol Cue Reactivity

**Figure 1:**
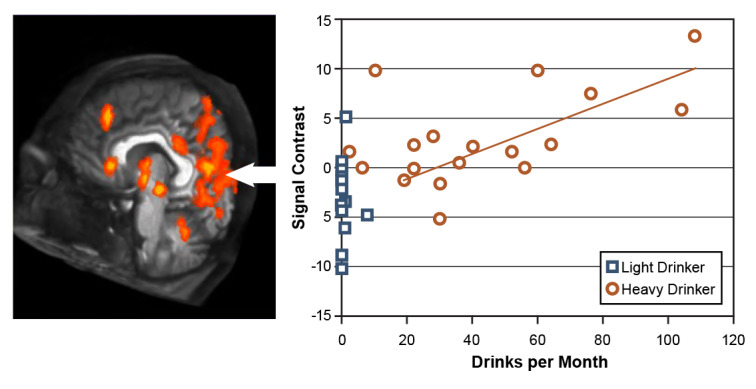
Response to alcohol pictures in youth with heavy versus light drinking Brains of youths who drank heavily activated strongly in response to seeing alcohol advertisements but showed little brain response to nonalcoholic beverage ads; this difference (i.e., signal contrast) was smaller in youth who drank lightly. The difference in brain response was greatest in adolescents with the highest consumption levels and was especially strong in the left hemisphere (positive affect), limbic, and visual cortex areas. *Source:* Tapert et al., 2003.^52^

Another set of studies demonstrated that youths who drank heavily exhibited greater brain activation while viewing alcohol advertisements^25^,^52^–^54^ than while viewing ads for nonalcoholic beverages.^52^ Adolescents are exposed to alcohol advertising materials on a daily basis in many countries. As studies in adults with AUD have shown atypical responses to alcohol-related materials,^55^ Tapert and colleagues used fMRI analyses to determine whether similar response patterns existed in adolescents who drink.^52^ The study included 15 adolescents ages 14 to 17 with AUD and 15 demographically similar adolescents who drank infrequently. The participants were shown pictures of alcoholic and nonalcoholic beverage advertisements during neuroimaging. Adolescents with histories of heavy drinking showed greatly enhanced neural activation while viewing the pictures of alcoholic beverages compared with pictures of nonalcoholic beverages. The extent of alcohol-related activation was greatest for those with the highest levels of monthly alcohol intake (see Figure 1). In contrast, youth with limited drinking histories showed similar levels of activation while viewing the two beverage picture types. These results demonstrated pronounced alcohol cue reactivity in heavy drinking teens, particularly in reaction to alcohol advertising materials.

## Factors Contributing to Adolescent Alcohol Use

### Age of Onset

Studies examining longer-term impacts of adolescent alcohol misuse have yielded mixed results. Some studies reported a maturing-out without significant consequences in adulthood, while others found ongoing effects on mental health, physical health, and social functioning, as well as higher levels of alcohol use and AUD.^56^ Analyses using data from the National Longitudinal Alcohol Epidemiologic Survey determined that 40% of those initiating alcohol use before age 15 were diagnosed with AUD at some point in their lives compared to only 10% of those who delayed the onset of drinking until age 21 or later.^57^

The first study of adolescents (ages 12 to 15 at baseline; *N* = 215) to assess the association between age of adolescent drinking onset and neurocognitive performance found that earlier age of drinking onset predicted poorer performance on tasks requiring psychomotor speed and visual attention. Similarly, an earlier age of onset of regular (weekly) drinking predicted poorer performances on tests of cognitive inhibition and working memory.^58^ This study suggested that early onset of drinking increased risk for subsequent neuropsychological dysfunction.

### Sex Disparities

Several studies have reported that the associations between alcohol and brain structure and function differ by sex, especially in adolescents engaging in binge drinking. While not conclusive across the literature, female adolescents who engaged in binge drinking appeared to show effects such as blunted activation in frontal, temporal, and cerebellar cortices compared to females who did not drink, whereas male adolescents who engaged in binge drinking showed the opposite activation pattern.^59^ Female adolescents ages 15 to 17 meeting criteria for AUD showed larger prefrontal cortex volumes than female controls, while male adolescents with AUD had smaller prefrontal cortex volumes than male controls.^60^ A similar finding was observed for white matter.

### Family History of AUD

Having a family member with AUD is associated with almost double the risk of initiating drinking in early adolescence.^57^ Using fMRI, Spadoni et al. observed greater neural activity during rest and reduced activity during an active baseline condition were linked to denser family history of AUD.^61^

### Mental Health Comorbidities

Adolescence is the peak time for both onset of substance misuse and emergence of mental illness, including anxiety disorders, bipolar disorder, major depression, eating disorders, and psychosis.^10^ The National Survey on Drug Use and Health (NSDUH) estimated that 20% of adolescents had a mental illness that persisted into adulthood.^2^ Moreover, adolescents with a past-year major depressive episode were more likely to be current binge alcohol users (6% vs. 4%).^2^ However, it remains unclear how comorbid mental health problems contribute to and exacerbate the neurobiological effects of alcohol misuse.^4^ Frontal and temporal cortical thinning may predict increased vulnerability to development of adolescent depression. In the NCANDA sample of 692 adolescents without a history of depression, the 101 youth who transitioned into depression were found at study baseline to have thinner cortices in the superior frontal cortex, precentral and postcentral regions, and superior temporal cortex, beyond effects attributable to age and sex.^62^,^63^

### Adverse Childhood Events

Childhood trauma and post-traumatic stress symptoms have been shown to confer increased risk for adolescent and adulthood AUD, mental illness, and physical health problems.^64^,^65^ Youth with trauma exposure showed thinner frontal cortices, and those with chronic post-traumatic stress disorder (PTSD) had smaller orbital frontal cortices^66^ and less superior posterior cortical and cerebellar gray matter volume.^67^ These observations indicate that trauma may be associated with structural brain aberrations.

**Figure 2:**
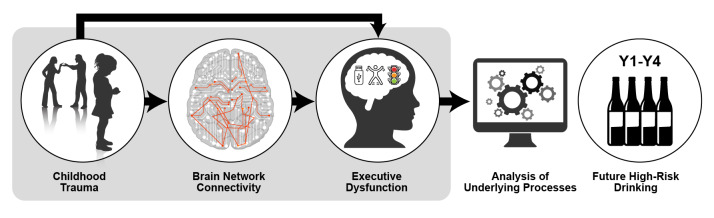
Model depicting how childhood trauma may lead to subsequent high-risk drinking *Note:* Y1–Y4, Year 1 through Year 4. *Source:* Silveira et al., 2020.^24^

NCANDA has also examined the relationship between childhood trauma and subsequent adolescent alcohol use.^68^ In a sample of 392 NCANDA participants, adverse childhood event history was linked to greater self-reported executive dysfunction spanning four annual follow-ups. Greater childhood trauma also was linked to less connectivity in sensorimotor and cognitive control networks (i.e., the bilateral dorsal anterior cingulate cortex, right anterior insula, right intraparietal sulcus, and bilateral pre- and postcentral gyri hub regions) at baseline. This reduced connectivity explained the relationship between executive dyscontrol and subsequent increased frequency of adolescent binge drinking (see Figure 2).^24^

### Poor Sleep

Sleep patterns change substantially during adolescence and emerging adulthood.^69^ Lack of sleep, going to sleep relatively late, and large weekend-weekday sleep differences all are risk factors for alcohol use in adolescents and young adults.^70^ Similarly, in the NCANDA sample, sleep difficulties in adolescence predicted later substance use problems.^71^ The reverse has also been seen, with acute and chronic alcohol intake altering sleep structure and electroencephalography patterns^72^ in older adolescents^73^ and adults.^69^ NCANDA will continue to longitudinally examine whether these changes remain evident into adulthood and how alcohol use influences sleep neurobiology.

### Use of Other Substances

Co-use of multiple substances may influence the relationship between alcohol use and neural integrity. For example, during a spatial working memory task, adolescents with co-occurring AUD and cannabis use disorder showed less inferior frontal and temporal neural activation but a greater medial frontal response compared to adolescents with AUD alone.^74^ Co-use of alcohol with cannabis also may adversely influence executive functioning.^75^ Given the high rates of co-occurring alcohol and other substance use during adolescence,^76^ future well-powered studies will benefit from detailed analyses of various combinations of substances of abuse on neural and neurocognitive outcomes.

## Recovery From Consequences of Adolescent Heavy Drinking

In adults with AUD, improvements in attention and concentration, reaction time, and memory are generally seen after 2 to 8 weeks of abstinence;^77^ however, executive functioning, processing speed, visuospatial, and verbal skills appear more resistant to recovery,^78^ and spatial processing deficits may persist for years.^79^ Younger adults tend to recover more quickly and completely than older adults (i.e., over age 50).^80^ As mentioned previously, preliminary evidence suggested that adolescent heavy drinkers showed greater response to alcohol cues,^54^ more emotional reactivity and poorer distress tolerance,^81^ and poorer visuospatial performance compared with adults.^82^ These effects remitted after a month of abstinence, indicating that some deficits are linked to alcohol intake and may be transitory. However, executive dysfunction^81^ and negative mood states^83^ did not remit within 4 weeks of abstinence, suggesting that these differences may have predated the onset of heavy drinking or may take more time to recover. As reported by Infante et al., cortical gray matter volume decreases were greater in proximity to reported drinking episodes in a dose-response manner, suggesting a causal effect and raising the possibility that normal growth trajectories may recover with alcohol abstinence.^40^ However, other studies have suggested that impaired visuospatial functioning following adolescent AUD persisted even after reduced levels of use.^84^

## Where Do the Data Lead Next?

Longitudinal studies with large, diverse, representative samples of youth and a range of detailed measures are key to helping understand the behaviors that convey disadvantages to adolescent and young adult development and outcomes. To date, a handful of large-scale multisite studies are being conducted to gain insight into the consequences of adolescents transitioning into and out of substance use. These include the largest long-term study of brain development in the United States, the Adolescent Brain Cognitive Development (ABCD) Study, which is currently underway; NCANDA; the IMAGEN study in Europe; the Pediatric Imaging, Neurocognition, and Genetics (PING) study; and the Lifespan Human Connectome Project (HCP) study. NCANDA has already been able to confirm impressions from prior smaller studies that adolescent heavy drinking appears linked to accelerated gray matter decline,^40^ disrupted functional connectivity,^30^ and reduced cognitive performance. Determining the degree to which these effects remit or persist with alcohol abstinence or reduced use will be a key next step in this line of work.
